# Ferroptosis in orthopedic implant integration after oncologic surgery

**DOI:** 10.3389/fonc.2026.1811290

**Published:** 2026-03-23

**Authors:** Pierre-Olivier Karbonowski, Gwan Yong Lim, Karolina Lach, Michał Skrzycki, Magdalena Mielczarek-Puta

**Affiliations:** 1 Student Scientific Club Explore, Chair and Department of Biochemistry, Faculty of Medicine, Medical University of Warsaw, Warsaw, Poland; 2 Chair and Department of Biochemistry, Faculty of Medicine, Medical University of Warsaw, Warsaw, Poland

**Keywords:** bone remodeling, ferroptosis, orthopedic oncology, reactive oxygen species, surface coatings, titanium implants

## Abstract

Primary bone cancer treatment often requires large bone resections followed by reconstruction using orthopedic implants, which are usually made of titanium (Ti). In oncologic patients, the bone-implant interface (BII) microenvironment is compromised by adjuvant therapies, such as chemotherapy and radiotherapy, which impair tissue repair and disrupt redox homeostasis. Despite high biocompatibility of Ti implants, their long-term success is challenged by the continuous release of degradation products Ti4+ ions and titanium dioxide (TiO2) nanoparticles, which promote chronic inflammatory and oxidative responses at the BII. Ferroptosis, an iron-dependent cell death characterized by lipid peroxidation primarily driven by an increase in reactive oxygen species, has emerged as an important contributor to understanding peri-implant pathology. Recent studies suggest that ferroptosis is associated with impaired osseointegration by inhibiting bone formation and accelerating bone resorption, leading to uncoupled bone remodeling, periprosthetic osteolysis and aseptic loosening. Therefore, modulating ferroptosis appears to be a potential therapeutic strategy. These strategies include implant surface modification with iron chelation or antioxidant coating, as well as pharmaceutical approaches targeting redox-regulatory pathways, such as glutathione peroxidase 4 (GPX4) and nuclear factor erythroid 2-related factor 2 (Nrf2) modulators. This molecular targeting presents a promising therapeutic avenue to preserve BII and potentially improve long-term implant survival in bone cancer patients. In this mini-review, we summarized the effects of ferroptosis on orthopedic implants, highlighting the potential of ferroptotic-targeting treatment to improve long-term implant outcomes in patients undergoing oncologic reconstruction.

## 1 Introduction

Primary bone cancer is a rare malignancy that arises from primitive mesenchymal cells. They can be subdivided into various subtypes, including chondrosarcoma, Ewing sarcoma, and osteosarcoma, which serve as the three most common primary bone cancers (1). Treatment for primary bone cancer predominantly relies on surgery to remove the tumor with chemotherapy and radiotherapy as supporting strategies (2). Reconstruction and functional restoration become essential aspects of patient management following surgical removal of the tumor.

Orthopedic implants are used to support damaged or deformed bones, joints, or cartilage, thereby improving the mobility and quality of life. Ti is the most commonly used material in orthopedic implants, while other materials include cobalt–chromium alloys, tantalum-based alloys, polymers such as ultra-high–molecular-weight polyethylene, and ceramics are also utilized (3).

Despite their benefits, continuous release of metallic particles and ions from Ti implants could potentially disturb redox homeostasis, thereby compromising their long-term success. The primary reason for implant failure is aseptic loosening, a pathological process characterized by a progressive bone loss at the BII, due to a chronic inflammatory response to implant degradation products (4). This pathological process emphasizes that BII is not an inert junction, but a biologically active microenvironment. However, these mechanisms alone do not fully explain the high rates of implant failure observed in oncologic patients compared with standard arthroplasty.

Unlike in standard arthroplasty, the BII in an oncological patient represents an even more hostile and biologically compromised microenvironment. The host bone is often weakened by neo-adjuvant or adjuvant therapies, including chemotherapy and radiotherapy, which impair tissue healing and vascularity. This “pre-sensitized” environment is particularly vulnerable to further stress (5, 6).

In addition to local tissue compromise, oncologic patients often exhibit systemic redox disturbances, including increased reactive oxygen species (ROS) level, altered iron metabolism, and antioxidant dysregulation (7). Together, this provides a microenvironment more susceptible to ferroptosis, an iron-dependent form of regulated cell death, increasingly implicated in peri-implant pathology. Therefore, understanding how ferroptosis contributes to impaired osseointegration has become essential in improving long-term outcomes in oncological patients.

## 2 Titanium degradation, mitochondrial ROS and ferroptosis initiation

Ferroptosis is an iron-dependent, regulated form of cell death distinct from apoptosis and other cell death modalities. Mechanistically, ferroptosis can be categorized into several interconnected pathways, including GPX4-dependent mechanisms, GPX4-independent antioxidant systems, dysregulation of iron metabolism, and excessive lipid peroxidation (8, 9). In GPX4-dependent ferroptosis, cystine will enter the cell through system Xc-, a dimer of SLC7A11 and SLC3A2, and is converted into cysteine through redox reaction. GPX4 with the help of glutathione as reducing substance, reduces polyunsaturated fatty acids (PUFA)-OOH into non-toxic PUFA-OH to prevent lipid peroxidation. Ferroptosis Suppressor Protein 1 (FSP1) axis serves as alternative method for ferroptosis defense mechanism without involvement of GPX4. In this pathway, FSP1 reduces CoQ10 to ubiquinol using NAD(P)H, helping to suppress lipid peroxidation. Other GPX4-independent pathway includes GTP cyclohydrolase 1 (GCH1)–Tetrahydrobiopterin (BH4) pathway and Dihydroorotate dehydrogenase (DHODH)–CoQH2 pathway, with the latter mainly act on mitochondrial ferroptosis defense (9). These pathways converge on a shared biological outcome characterized by disrupted redox homeostasis and uncontrolled lipid peroxidation.

Mitochondria contribute to ferroptosis through ROS generation and metabolic regulation of redox. However, ferroptotic cell death can also be driven by non-mitochondrial lipid peroxidation cascades and iron-dependent radical reactions. In the peri-implant context, most evidence linking mitochondrial ROS to ferroptosis is extrapolated from broader bone and oxidative stress models, whereas direct mitochondria-targeted rescue studies at the bone–implant interface remain limited (10).

Ti implants are highly compatible but not biologically inert. Over time, implant degradation causes release of particles, which trigger inflammatory responses, leading to oxidative cell death. The degradation of Ti implants is a multifactorial process which is called tribocorrosion (11). A central component of this degradation is mechanical wear, due to fretting. This friction causes the removal of the protective layer, like TiO2, which forms the implant’s surface (12). Once the protective layer is breached, the underlying metal is exposed to body fluids like synovial fluid, which is rich in chloride ions. This accelerates degradation of Ti, further speeding up the breakdown (13).

Another important factor is the inflammatory microenvironment, triggered by corrosion or pre-existing inflammation. It creates a highly acidic environment which is rich in ROS and subsequently initiates a vicious cycle that accelerates the corrosion rate (13). These combined pathomechanisms release degradation products, primarily Ti4+ ion and TiO2 nanoparticles (NPs) into the surrounding tissues. These Ti4+ ions and TiO2 NPs are recognized as foreign bodies by the immune system, especially by macrophages.

Macrophages engulf titanium nanoparticles, and this cytotoxic phagocytic process induces mitochondrial dysfunction, leading to excessive ROS production. At the same time, Ti NPs activate the nuclear factor-κB (NF-κB) signaling pathway, which is one of the main regulators of inflammation. TiO2 NPs also physically damage the lysosome in macrophages, triggering the formation of NLRP3 inflammasome by destroying the lysosome (14, 15).

Although ROS are part of the normal inflammatory response, their overproduction spills into the peri-implant microenvironment, increasing oxidative stress at the bone–implant interface. This impairs osteoblast function, promotes osteoclast activation, disrupts bone remodeling, and creates a pro-oxidative, iron-dysregulated environment that favors ferroptosis in bone cells (16–18). Titanium-induced oxidative stress has been accompanied by suppression of GPX4 expression, resulting in impaired defense against lipid peroxides and increased susceptibility to cell death (3, 19).

Beyond implant-induced oxidative stress, oncologic patients often exhibit impaired iron metabolism and redox homeostasis, which predispose them to ferroptosis. Cancer cells usually have higher demand for iron compared to normal cells, in order to support their rapid proliferation. This is accomplished by increased expression of transferrin receptors and ferritin turnover. This results in expanded labile iron pool and enhanced Fenton reaction, facilitating lipid peroxidation. Malignancy-associated chronic inflammation produces pro-inflammatory cytokines like interleukin-6 (IL-6), which upregulate hepcidin expression and further alter iron distribution.

In addition to alternations by cancer itself, radiotherapy and chemotherapy can further amplify oxidative stress and iron metabolism. Methotrexate, as one of the cornerstone chemotherapy drugs used in clinical practice, has shown to increase oxidative stress and may sensitize cells to ferroptotic injury (20). While ferroptosis may occur in standard arthroplasty, these systemic alterations in oncologic patients may lower the threshold for oxidative damage and increase ferroptosis risk at the bone–implant interface.

## 3 Ferroptosis-mediated uncoupling of bone remodeling

Successful osseointegration depends on a robust population of functional osteoblasts and their progenitors, mesenchymal stem cells (MSCs), which migrate to the BII, proliferate, differentiate, and produce a mineralized extracellular matrix (21). Emerging evidence indicates ferroptosis as a key pathological mechanism that leads to implant failure in oncologic settings. The failure is coordinated by a two-pronged process: 1) suppression of bone formation via ferroptotic death of osteogenic cells, and 2) prolongation of a pro-inflammatory, osteolytic environment by immune activation (22, 23).

Bone remodeling is severely compromised due to two major triggers: iron dysregulation and oxidative stress arising from adjuvant therapies, such as radiation therapy, as well as implant debris (24, 25). When osteoblasts and MSCs undergo ferroptosis, their osteogenic capacity is severely impaired. This not only causes cellular death, but also actively suppresses osteogenic differentiation processes. Ferroptotic stress downregulates the expression of key osteogenic genes, including Run-related transcription factor 2 (Runx2), Osterix (Osx) and Nrf2 (21). In the absence of these signaling pathways, MSCs fail to differentiate into mature osteoblasts and compromise their ability to secrete or mineralize the bone matrix, resulting in defective bone formation at the BII.

Under physiological conditions, macrophages transition from a pro-inflammatory to a pro-healing phenotype; however, within a ferroptosis-associated, ROS-rich peri-implant microenvironment, this transition is disrupted, leading to sustained inflammatory activation. Persistent pro-inflammatory macrophages produce high levels of inflammatory signals and release cytokines such as tumor necrosis factor-α (TNF-α), interleukin-1β (IL-1beta) or IL-6 (26). Meanwhile, cells undergoing ferroptosis release damage-associated molecular patterns (DAMPs), which further promote the activation of surrounding immune cells, and consequently enhance the inflammatory response (27). Research on inflammatory bone loss shows that ferroptotic macrophages secrete significantly higher levels of TNF-alpha, which disrupts the Nrf2-dependent antioxidant pathway within the macrophages (28). Within this inflammatory context, pro-inflammatory cytokines such as TNF-α and IL-1β act as potent stimulators of osteoclastogenesis. They promote expression of RANKL, which is the essential signal that causes osteoclasts’ precursors to fuse and mature into bone-resorbing osteoclasts, leading to peri-implant osteolysis (29).

**Figure 1:**
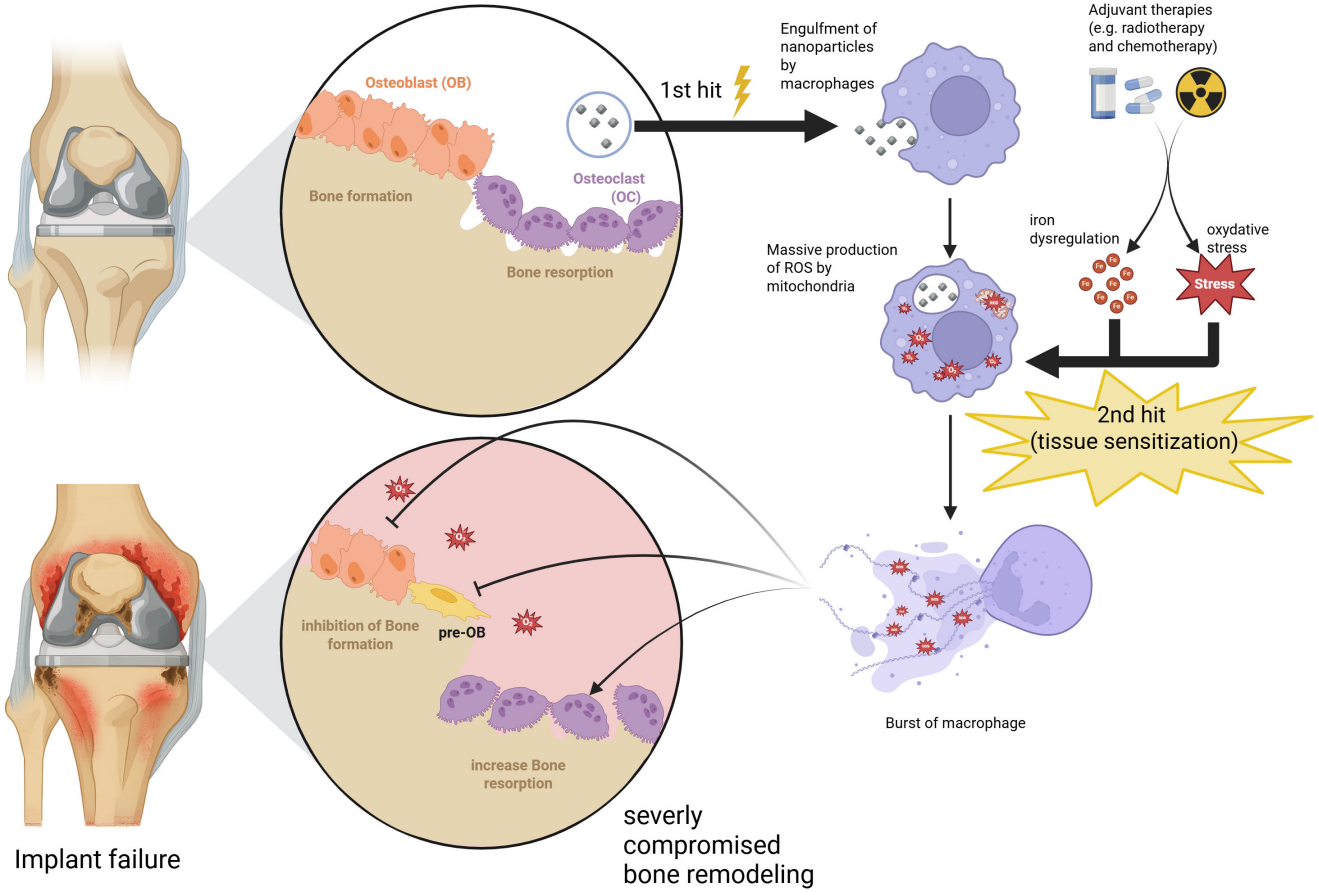
Proposed double-hit pathophysiological framework of implant failure in oncological patients (30).

With bone being actively destroyed and no new bone being formed to replace it, a gap or a fibrous tissue layer is formed between the bone and the implant. This leads to mechanical instability, micromotion and aseptic loosening (**Figure 1**).

## 4 Potential therapeutic modulation of ferroptosis in titanium implants

Given the central role of iron as a catalyst of oxidative damage via the Fenton reaction, one of the potential strategies is to reduce its local bioavailability. Iron chelation therapy is used to bind and sequester excess iron and shows promising therapeutic modulations of ferroptosis in orthopedic implants and promoting osseointegration. Therapeutic approaches are categorized into: A) ferroptosis-targeted (iron chelation, radical traps, GPX4/SLC7A11/Nrf2 support, mitochondria-targeted antioxidants) and B) downstream bone remodeling therapies.

A promising therapeutic strategy is the use of small-molecule and iron chelators that coat the Ti implant. This can be illustrated by a new caffeic acid (CA) nanospheres and deferoxamine (DFO) film on the Ti implant surface. Polyphenol (caffeic acid) coating provides localized antioxidant activity, scavenges ROS, activates Kelch-like ECH-associated protein 1 (KEAP1)/Nrf2 signaling, and could reduce lipid peroxidation, consequently diminishing ferroptotic signaling in mesenchymal stem cells. DFO film, under high oxidative stress, could chelate excess Fe and lower the labile iron pool locally, thereby iron chelation-mediated lipid peroxidation. In an osteoporotic implant model, this dual coating raises GPX4/SLC7A11 expression, reduces lipid ROS and markedly improves bone formation compared to unmodified Ti (31–34).

Systemically administered mitochondria-targeted antioxidants represent a promising potential therapy. They specifically accumulate within the mitochondrial matrix, the cells’ primary site of ROS production and could effectively inhibit the peroxidative chain reactions essential for ferroptosis (35, 36).

Beyond radical trapping, natural compounds such as urolithin A, have been shown to systematically alleviate Ti NPs-induced osteoblastic ferroptosis and osteolysis-induced bone loss as well as to promote osteoblast differentiation in an animal model (37). Other natural compounds, that have traditionally been administered systemically, like maresin1, silymarin, and xanthohumol protect osteoblasts from ferroptosis (38).

Also emerging ferroptosis inhibitors, such as liproxstatin-1 and ferrostatin-1, show strong, localized cytoprotective effects in oxidative microenvironments and could represent potential adjunct local therapies (39).

A range of pharmaceutical agents has shown significant anti-osteolytic efficacy. These include established therapies such as bisphosphonates, selective estrogen receptor modulators (SERMs, e.g., raloxifene), the anti-RANKL antibody denosumab, and parathyroid hormone analogs (e.g., teriparatide, abaloparatide). Newer agents, notably sclerostin modulators (e.g., romosozumab, blosozumab), are also emerging in clinical practice. These drugs are administered systemically and despite their efficiency, the clinical application remains restricted by long-term side effects, like cardiovascular, renal and hepatic toxicities, as well as risk of malignancy and osteonecrosis (40).Another possibility is to fortify defense mechanisms by modulating upstream signaling pathways. Metformin can systematically activate the AMP-activated protein kinase (AMPK) pathway and has been shown to strongly inhibit ferroptosis by triggering the Nrf2 signaling pathway. Nrf2 functions as a central regulator of the cellular antioxidant response, promoting the transcription of multiple cytoprotective genes, including GPX4, thereby potentially exerting protective effects that reduce osteolysis and aseptic loosening (41).

Notably, ferroptosis-targeted strategies such as iron chelation coatings, antioxidant surface modifications, and ferroptosis inhibitors are amenable to localized delivery at the BII, whereas conventional bone remodeling agents are primarily administered systemically and act downstream of ferroptotic signaling (**Table 1**).

**Table 1:** Possible ferroptosis targeting strategies with mechanism of action and specific effects and outcomes.

Treatment/Agent	Mechanism of action	Specific effects & outcomes	References
Mitochondria-targeted Antioxidants	Accumulates specifically within the mitochondrial matrix.	Effective at inhibiting the peroxidative chain reactions that are essential for ferroptosis.	(25, 35)
Polyphenol Coatings (e.g., CA Nanospheres)	Provides antioxidant activity and scavenges ROS.	Activates KEAP1/Nrf2 signaling, reduces lipid peroxidation, and diminishes ferroptotic signaling in mesenchymal stem cells.	(31, 32)
Iron Chelation Coatings (e.g., DFO film)	Binds and sequesters excess iron, reducing local bioavailability.	Lowers the labile iron pool locally and prevents iron chelation-mediated lipid peroxidation.	(33)
Urolithin A	Directly activates GPX4, the cell’s main enzymatic defense against lipid peroxidation.	Alleviates Ti nanoparticle-induced osteoblastic ferroptosis, reduces osteolysis-induced bone loss, and promotes differentiation.	(37)
Liproxstatin-1 & Ferrostatin-1	Radical trapping antioxidant	Shows strong cytoprotective effects in oxidative microenvironments.	(39)
Bisphosphonates	Reduces osteoclast proton production, suppresses osteoclast maturation and promotes osteoclast apoptosis	Decreases bone resorption, established anti-osteolytic therapy, though long-term utility may be constrained by adverse reactions (AR). Possible AR: hypocalcemia, hypophosphatemia osteonecrosis, esophageal inflammation	(40)
Denosumab	Anti-RANKL antibody, prevents osteoclast maturation	Decreases of bone resorption, established anti-osteolytic therapy. AR: severe hypocalcemia, hypersensitivity, osteonecrosis of the jaw	(40)
Sclerostin Modulators (e.g.,Romosozumab, Blosozumab)	Modulates sclerostin	Promotes bone formation,emerging agents in clinical practice for bone loss management. AR: arthralgia, headache, myocardial infarction, stroke, cardiovascular death	(40)
Metformin	Activates the AMPK pathway, which triggers the Nrf2 signaling pathway, upregulates protective genes (including GPX4)	Reduces osteolysis and aseptic loosening. AR: gastro-intestinal distress, reduced appetite, weight changes, headache, vitamin B12 deficiency, lactic acidosis, hypoglycemia	(41)
SERMs (e.g., Raloxifene)	Selective estrogen receptor modulator. Inhibits osteoclasts, produces osteoprotegerin	Reduces bone turnover, used as an established therapy for anti-osteolytic efficacy. Possible AR: venous thromboembolism, cataract, hot flashes	(42)
PTH Analogs (e.g.Abaloparatide)	Parathyroid hormone analogs, increases osteoblastic activity	Promotes bone formation, established therapy showing anti-osteolytic efficacy. Possible AR: paresthesia, hypocalcemia, headache, hypercalcemia, nausea, hypoesthesia	(43)

The table includes not only ferroptosis-targeting agents but also compounds with direct effects on bone metabolism.

## 5 Discussion

Traditionally, implant failure is attributed to mechanical wear and non-specific inflammation. However, the concept of ferroptosis provides a potential molecular explanation that helps connect several previously described pathophysiological processes. By linking metallic degradation products to cellular death, ferroptosis may offer a molecular explanation for the extent of tissue loss and implant failures observed in affected patients.

The framework is particularly relevant in oncological patients, whose BII differ fundamentally from those of patients who underwent standard arthroplasty. In this context, implant failure appears to result from a compounded pathological synergy - a double-hit mechanism, a hypothesis supported by convergent preclinical findings. While the first hit stands for localized Ti nanoparticles and chronic ROS release at the BII (19), the second hit emerges from adjuvant therapies used in oncological patients (44). Ferroptosis may therefore represent a key contributing pathomechanism of implant failure, triggered by a threshold of oxidative distress that healthy tissues might otherwise tolerate.

In the ferroptotic model, the uncoupling of bone remodeling is likely not only an imbalance of signals, but potentially a result of the sustained “pro-ferroptotic” inflammatory state of macrophages and death of the osteogenic lineage (45). By viewing the BII as a dynamic site of regulated cell death, we may change the focus of managing inflammation to actively protecting cellular viability and preserving redox integrity.

The consequences for osteointegration appear to be substantial, with potential inhibition of bone formation and acceleration of bone resorption. This decoupling of bone remodeling may act as the fundamental mechanism of osteolysis and aseptic loosening. The identification of ferroptosis in this process may provide a precise molecular target for managing this inflammation (46).

Conceptualizing ferroptosis as one of the contributing mechanisms in implant failure among oncological patients positions it as an actionable biological target. It may justify a shift toward coated orthopedic implants. Whether through local iron sequestration or fortification of the Nrf2/GPX4 axis, therapeutic modulation presents a potentially promising strategy to stabilize the BII and to help ensure long-lasting implant successes (47).

Taken together, these observations suggest that ferroptosis may not simply be a downstream consequence of oxidative damage but may also be an important coordinator of the long-term biological response to Ti implants in oncologic settings.

Nevertheless, ferroptosis does not act in isolation within the peri-implant microenvironment. Apoptosis and other forms of regulated cell death likely coexist and intersect with ferroptotic signaling, thereby modulating cell survival, inflammatory signaling, and tissue remodeling at the BII (48, 49). Apoptosis and pyroptosis are also involved in peri-implant inflammation by releasing pro-inflammatory cytokines such as IL-1β. These mechanisms disrupt bone homeostasis and promote osteolysis. Recent studies suggest that a bidirectional regulatory mechanism between ferroptosis and classical inflammation-related signaling pathways, like NF-κB, inflammasome or mitogen-activated protein kinase (MAPK) signaling pathways, exists (50).

Lately, natural compounds have been reported to modulate ferroptosis, especially in cancer patients. These effects occur through mechanisms involving redox regulation, iron metabolism, and lipid peroxidation. Despite these promising approaches, effective clinical translation remains challenging (51). Future research should focus on natural compounds that demonstrate cytotoxic effect at physiological concentrations, as this is essential for translating the pre-clinical findings into clinical setting. Targeting ferroptosis may hold the potential to improve long-term implant survival and, consequently, the quality of life in patients with bone malignancy.

## 6 Conclusion

The successful integration of orthopedic implants following oncologic surgery remains a significant clinical challenge compared to standard arthroplasty. This review proposed that ferroptosis may represent a mechanistic explanation for Ti degradation, mitochondrial reactive oxygen species generation, and iron dysregulation at the bone–implant interface, processes that contribute to implant failure in oncological patients. As the number of oncologic reconstructions continues to increase, ferroptosis provides a conceptual framework for reducing the risk of implant failure, helping to shape future strategic approaches. The development of strategies that focus on the modulation of iron homeostasis and the inhibition of ROS represents promising therapeutic potential following oncologic surgeries.

## Glossary

AMPK: AMP-activated protein kinase
AR: Adverse reaction
BH4: Tetrahydrobiopterin
BII: Bone-implant interface
CA: Nanospheres
DAMPs: Damage-associated molecular patterns
DFO: Deferoxamine
DHODH: Dihydroorotate dehydrogenase
FSP1: Ferroptosis Suppressor Protein 1
GCH1: GTP cyclohydrolase 1
GPX4: Glutathione peroxidase 4
IL-1β: Interleukin-1 Beta
IL-6: Interleukin-6
KEAP1: Kelch-like ECH-associated protein 1
MAPK: Mitogen-activated protein kinase
MSCs: Mesenchymal stem cells
NF-kB: Nuclear factor-κB
NP: Nanoparticles
Nrf2: Nuclear factor erythroid 2-related factor 2
Osx: Osterix
PUFA: Polyunsaturated fatty acids
RANKL: Receptor Activator of Nuclear Factor kappa B Ligand
ROS: Reactive oxygen species
Runx2: Run-related transcription factor 2
TI: Titanium
TiO2: Titanium dioxide
TNF: Tumor necrosis factor
